# A Textbook of Diseases of the Eye

**Published:** 1903-02

**Authors:** 


A TEXTBOOK OF DISEASES OF THE EYE. A Handbook of Ophthalmic Practice for Students and Practitioners. By G. E. DE SCHWEINITZ, A. M., M. D., Professor of Ophthalmology in the University of Pennsylvania. Fourth edition, revised, enlarged and entirely reset. Octavo volume of 773 pages, with 280 text-illustrations and 6 chromo-lithographic plates. Philadelphia and London: W. B. Saunders & Co. 1902. [Cloth, $5.00 net; sheep or half-morocco, $6.00 net.]

The fourth edition of this standard work on the eye shows a complete revision; over one hundred pages have been added to the text, together with nearly fifty additional illustrations. Metastatic gonorrheal conjunctivitis; the so-called holes in the macula; grill-like keratitis; divergence paralysis, convergence paralysis and other new chapters will prove of interest and greatly enhance the value of the work. The newer silver salts are mentioned in connection with the diseases in which they are indicated. The only disappointment will be the treatment of granular ophthalmia, for de Schweinitz still advocates the use of sulphate of copper and strong bichloride, or nitrate of silver solutions. R. H. S.

